# Macular neovascularization and polypoidal choroidal vasculopathy: phenotypic variations, pathogenic mechanisms and implications in management

**DOI:** 10.1038/s41433-023-02764-w

**Published:** 2023-10-06

**Authors:** Chui Ming Gemmy Cheung

**Affiliations:** 1 Singapore Eye Research Institute, Singapore National Eye Centre, Singapore, Singapore; 2 Ophthalmology & Visual Sciences Academic Clinical Program (Eye ACP), Duke-NUS Medical School, Singapore, Singapore

**Keywords:** Diseases, Eye diseases

## Abstract

Advances in imaging have led to improved ability to characterize variations in clinical sub-phenotypes of macular neovascularization (MNV) in Age-related macular degeneration (AMD). Polypoidal choroidal vasculopathy (PCV) was initially described based on characteristic features observed in indocyanine green angiography (ICGA) and was thought to be a distinct entity from AMD. However, subsequent careful observations based on confocal scanning laser ophthalmoscopy-based ICGA, optical coherence tomography (OCT) and OCT angiography have led researchers to appreciate similarities between PCV lesion and type 1 MNV in typical neovascular AMD. Concurrently, clinical trials have shown that anti-VEGF monotherapy can achieve favourable visual outcome in the majority of eyes with PCV. These learnings have led to a shift in the way PCV is managed over the past decade. Recent studies have supported the use of non-ICGA based imaging modality to screen for PCV and the adoption of anti-VEGF monotherapy as initial therapy for PCV. A focus of recent research has been in the understanding of the role of choroidal alterations in the pathogenesis of PCV. The concept of pachychoroid in leading to outer retinal ischemia has garnered increasing support. Future research in this area should evaluate the potential of choroidal morphology in guiding personalized therapy in PCV.

## Abstract (zh)

影像技术的进步提高了追踪老年黄斑变性(AMD)中黄斑新生血管(MNV)亚临床表型变化的能力。息肉状脉络膜血管病变(PCV)最初是根据吲哚菁绿血管造影(ICGA)观察到的特征描述的, 被认为是AMD亚型。然而, 随后基于共聚焦扫描激光检眼镜的ICGA、光学相干断层扫描(OCT)和OCTA的观察使研究人员认识到典型新生血管性AMD中PCV病变与1型MNV 的相似性。同时临床试验表明, 抗VEGF单药治疗可在大多数PCV患眼中获得良好的视力获益。在过去十年这些经验导致PCV的治疗方式发生了转变。最近的研究支持使用非ICGA的成像方式筛查PCV, 并采用抗VEGF单药治疗作为PCV的初始疗法。最近的研究重点是了解脉络膜的病理变化在PCV发病机制中的作用。导致视网膜外层缺血的肥厚性脉络膜的概念得到越来越多的支持。该领域未来的研究应评估脉络膜形态学来指导PCV个体化治疗中的潜力。

## Background

The recognition of sub-phenotypes may have important implication for treatment strategies and outcomes in many conditions, including macular neovascularization (MNV). For example, photodynamic therapy (PDT) was most effective in eyes with pure classic and predominantly classic choroidal neovascularization (CNV) [1]. Hence detailed evaluation of the fluorescein angiography was important in during the PDT era. Anti-VEGF therapy, on the other hand, is highly effective for macular neovascularization in the context of age-related macular degeneration, and has been the standard of care for almost two decades. In the MARINA and ANCHOR trials, about 95% of eyes treated with ranibizumab avoided losing 15 letters or more, regardless of classic or occult lesion subtype [2, 3]. With the advent of multimodal imaging incorporating indocyanine green angiography (ICGA) and optical coherence tomography (OCT), clinicians are increasingly vigilant in recognizing additional subtypes of MNV such as type 3 MNV and polypoidal choroidal vasculopathy (PCV) (aka aneurysmal Type 1 MNV) [4]. Differentiating these sub-phenotypes is important in view of the potential implication on therapy. In addition, observations from studying these phenotypic variations can help the understanding of differences in underlying pathogenic mechanisms within a complex condition, reflecting both genetic and environmental influences.

## Phenotypic variation

### PCV: distinct entity?

PCV was first described in the 1990s and was initially thought to be a vascular disease of the choroid [5–7]. Differences in clinical features and demographics between PCV and typical nAMD have been described by many researchers. Eyes with PCV have a propensity to present with macular haemorrhage, causing large serosangiounous detachments, and even break-through vitreous haemorrhage in severe cases [8–11]. A subretinal orange nodule may be detected on clinical examination. Importantly, drusen, widely considered a hallmark of AMD, are less frequently present in patients with PCV, while a history of past central serous chorioretinopathy has been described [12–15]. Differences in demographic features include a higher prevalence of PCV in Asian populations and in males, and affecting a younger age group compared to typical nAMD. In clinic-based case series presenting with exudative AMD, PCV contributes to up to 60% in Asian population but only up to 20% in white European patients [16–18]. PCV also affects patients with Hispanic and Afro-Caribbean descent. However, most of the published data have focused on comparison between patients from Asians versus European descent. A preponderance of peripapillary and haemorrhagic presentation has been described among patients of Afro-Caribbean descent. There were few histopathology studies to refer to, and these have reported inconsistent findings. Reports that suggested PCV represent a distinct entity found the presence of hyalinization of choroidal vessels like arteriosclerosis and absence of VEGF staining in PCV specimens [19]. In contrast, other groups have observed the presence of proliferating fibrovascular tissue positive for VEGF [20–23].

Eyes with PCV typically exhibit occult pattern of leakage on fluorescein angiography (FA). Therefore, FA appearance is rarely helpful in differentiating PCV from type 1 MNV [12, 24, 25]. Indocyanine green angiography (ICGA) has been the gold standard for diagnosing PCV, in which the polyp is the most characteristic feature which typically appears as nodular hyperfluorescence appearing during the early-phase ICGA, sometimes exhibiting pulsatility, and becomes brighter in the mid-phase ICGA with pooling of the ICG dye [26, 27]. The polyp(s) can be seen to arise from the terminus of a branching vascular network (BVN). However, visualization of the BVN with fundus-camera based ICGA had been challenging due to low contrast. With confocal scanning laser ophthalmoscopy (cSLO)-based ICGA, a BVN can be visualized in almost all PCV complex [28, 29].

The natural history of PCV has been reported to be more variable than that of typical nAMD. Early reports suggested some eyes with PCV may have a relatively benign prognosis, which contrasts with that of typical nAMD. In 2002, Uyama reported a series of 14 eyes with PCV untreated for at least 2 years, 50 % of whom remained stable and maintained vision of 20/30 or better [30]. Spontaneous involution of polyps may also occur. Before the advent of anti-VEGF, treatment strategies for PCV targeted closure of the polyp with various occlusive therapies including focal laser and photodynamic therapy (PDT) [31–33]. Resolution of haemorrhage and stabilization of vision could frequently be achieved with these strategies. In fact, responses to PDT appeared more favourable in PCV eyes compared to typical nAMD eyes and require fewer retreatments [34, 35].

With the publication of MARINA and ANCHOR, anti-VEGF had taken over PDT as the standard of care in typical nAMD around the world. The DENALI study further showed no benefit of combining intravitreal ranibizumab with PDT in typical nAMD [36]. However, early experience of off-label intravitreal bevacizumab (IVB) in PCV from Asia reported variable responses, and highlighted while exudation was controlled, polyp closure was not frequently seen [37–39]. Other authors have reported refractory cases to anti-VEGF turned out to be PCV and highlighted the importance to recognize these cases and perform ICGA to confirm the correct diagnosis [40]. A clinical study performed at the Singapore National Eye Center between 2010 and 2014 showed that anti-VEGF monotherapy was only used in 15% of eyes with PCV while it was used for the vast majority of eyes with typical nAMD [41]. In 2012, the EVEREST study was published. Despite its small sample size of 60, it was highly impactful, as it was the first randomized controlled trial (RCT) in anti-VEGF in PCV. The EVEREST study compared PDT alone, PDT combined with intravitreal ranibizumab (IVR), and IVR monotherapy in 60 participants in a randomized design over 6 months, and reported higher polyp closure (77.8% and 71.4% versus 28.6%, *p* < 0.01) in the two PDT treatment arms compared to IVR alone [42]. However, visual gains were similar in the 3 groups (10.9 letters, 7.5 letters and 9.2 letters, respectively), and the sample size for this study was insufficient to detect statistically significant differences in BCVA changes.

These early findings led to the impression of PCV being a distinct entity to typical nAMD, with different underlying pathogenic mechanisms and requiring different diagnostic and therapeutic approaches. Specifically, the importance of performing ICGA and the need for PDT were highly emphasized [43]. In fact, in response to the publication of the CATT study in 2011, our group published a correspondence to raise the concern that those results may not apply to the Asian population because of the higher prevalence of PCV [44], and the authors agreed with this assertion.

### PCV as a subtype of AMD

Advances in genotyping and phenotyping have generated a number of important novel findings in PCV which led to a shift towards considering PCV as a subtype of AMD, and not a separate entity. Studies in systemic and genetic risk factors have reported similar findings in PCV and in typical nAMD. Imaging studies have highlighted the similarity between the BVN in PCV and a type 1 MNV. Concurrently, accumulation of clinical experience of anti-VEGF monotherapy to treat PCV further supports this evolution.

Genetic studies of AMD have identified a number of susceptibility single nucleotide polymorphisms (SNPs) in genes involved in the complement cascade, inflammatory pathway, extracellular matrix regulation and lipid metabolism [45–49]. The International AMD Genomics Consortium identified 34 AMD loci based on a large collection of over 16 000 cases and controls respectively of European ancestry [50]. Many of these AMD-associated loci have also been associated with PCV, although studies for PCV have been limited to smaller scale candidate gene approaches at selected SNPs. These studies reported significant associations in PCV in a number of AMD-associated loci, with CFH and ARMS2-HTRA1 being the most strongly associated with PCV [48]. Our group published a meta-analysis of association in a total of 1062 PCV patients, 1157 tAMD patients and 5275 controls of East Asian descent from the Genetics of AMD in Asians (GAMA) Consortium at the 34 known AMD loci. Eight AMD-associated gene/loci were significantly associated with PCV, including *ARMS2-HTRA1, CFH, C2-CFB-SKIV2L, CETP, VEGFA, ADAMTS9-AS2, TGFBR1*, and *COL4A3*. These similarities suggest that PCV is genetically highly correlated with typical nAMD. Further studies with candidate gene analysis approach reported shared SNPs for both AMD and PCV in genes involved in angiogenesis pathway (e.g., vascular endothelial growth factor (VEGF), placental growth factor (PGF), and ANGPT2).

The advent of spectral-domain optical coherence tomography (SD-OCT) had revolutionized the retinal practice. The ability to accurately detect and quantify intra- and sub-retinal fluid has become the gold standard for assessing anatomical response to anti-VEGF therapy. In addition, the ability to appreciate changes in the contour of the retinal pigment epithelium (RPE) band had major implication in the understanding of MNV sub-phenotypes. Type 1, 2 and 3 MNV can be readily differentiated based on the relation of the neovascularization to the RPE [51]. In this respect, with SD-OCT, researchers have been able to identify the BVN of the PCV complex consistently at a level between an elevated RPE anteriorly and the thin hyperreflective line representing the outer portion of Bruch’s membrane posteriorly [52]. Furthermore, using OCT angiography (OCTA), our group have reported persistence of flow within the BVN in >90% of eyes following treatment with anti-VEGF monotherapy or combined with PDT [53]. This persistent flow was present even in eyes with dry retina. These findings strongly support the notion that the BVN is not intrachoroidal but represents a form of neovascularization similar to a type 1 MNV, although perhaps with lower exudative activity [54].

Two important phase 3 randomized controlled trials were published in 2017-2018. These were the first large scale reports of using anti-VEGF monotherapy in PCV. The EVEREST II study expanded its hypothesis from the first EVEREST study to investigate whether combination therapy (IVR + PDT) was superior to IVR monotherapy [55]. The primary endpoint at month 12 showed superiority of the combination arm, gaining 8.3 letters compared to 5.1 letters respectively. The PLANET study, on the other hand, compared intravitreal aflibercept (IVA) monotherapy to IVA plus rescue PDT, and reported IVA monotherapy was non-inferior to IVA plus rescue PDT, gaining 10.7 and 10.8 letters, respectively [56]. Only about 15% of participants met the rescue criteria. It is important to point out that the vast majority of eyes avoided losing 15 letters or more- a finding similar to that seen in the MARINA and ANCHOR trials. Based on these RCTs, a major review and management recommendation was published in 2018, in which both anti-VEGF monotherapy and combination with PDT are considered acceptable first line therapy.

While polyp closure had been an important indicator for treatment success, we learned from the EVEREST II and PLANET studies that achieving a dry retina on OCT may be a more important anatomic endpoint than polyp closure on ICGA as good functional outcome can be achieved even in the presence of polyps, as long as they are not leaking (inactivated). This new concept can be derived from the impressive visual acuity gains in the PLANET study despite angiographic closure rate of only 44%.

Subsequent studies evaluating other anti-VEGF agents used as monotherapy in PCV have also reported favourable response, further supporting that PDT is not essential for PCV as the first line therapy. Brolucizumab has been shown to be non-inferior to aflibercept in the HAWK and HARRIER trials for nAMD. A subgroup analysis of patients diagnosed with ICGA-defined PCV was performed on Japanese participants from the HAWK trial. In this subgroup analysis, both treatment groups had similar mean changes in BCVA (+11.4 letters versus +11.1 letters) at week 96, with 68% of eyes with PCV treated with brolucizumab achieving 12-weekly dosing intervals [57]. Patients in the brolucizumab group had lower proportion of eyes with IRF and/or SRF at week 96 (12.8 % versus 16.7%). The PULSAR study (NCT04423718), a randomized control study, assessed the efficacy of 8 mg intravitreal aflibercept (IVA) every 12 or 16 weeks compared to 2 mg IVA every 8 weeks in participants with nAMD. A total of 297/1009 (29.4%) participants underwent ICGA, of which 141 had PCV. The BCVA gains in this subgroup analysis showed similar gains across treatment groups (+9.3, +8.5 and +9.5 letters in the 8 mg IVA every 12 weeks, 8 mg IVA every 16 weeks and 2 mg IVA every 8 weeks arms respectively at 48 weeks. The proportion of participants that were maintained on >12 weeks’ intervals was also similar to the overall nAMD cohort.

Overall, the findings from these studies in risk factors, imaging features and treatment outcome have led to a shift in mind-set in the management of PCV to that closer to typical nAMD [9, 58, 59]. The primary goal of therapy has shifted to achieving best visual outcome while minimizing treatment burden. Achieving polyp closure, which has been the previous emphasis, was now placed as secondary goal. This is a key paradigm shift in the management consideration of PCV. Accordingly, the treatment modality has shifted from previously occlusive and ablative therapy, targeting at angiographic closure, to control of disease activity guided by OCT to deliver functional outcome. Concurrently, anti-VEGF regimen has shifted from PRN previously to T&E.

## Evolution in management

The awareness of PCV has increased over the past two decades, even outside of Asia, where the prevalence is less common. The prevalence of PCV among patients presenting with exudative AMD has been estimated to be up to 20% in non-Asians, although it is believed to be somewhat underestimated as ICGA is not routinely performed [16–18]. As such, two highly clinically relevant questions are frequently posted by clinicians outside of Asia. First, how critical is it to perform ICGA to diagnose PCV? Second, how differently should PCV be managed compared to typical nAMD?

### Diagnosis- how critical is it to perform ICGA to diagnose PCV?

**Fig. 1:**
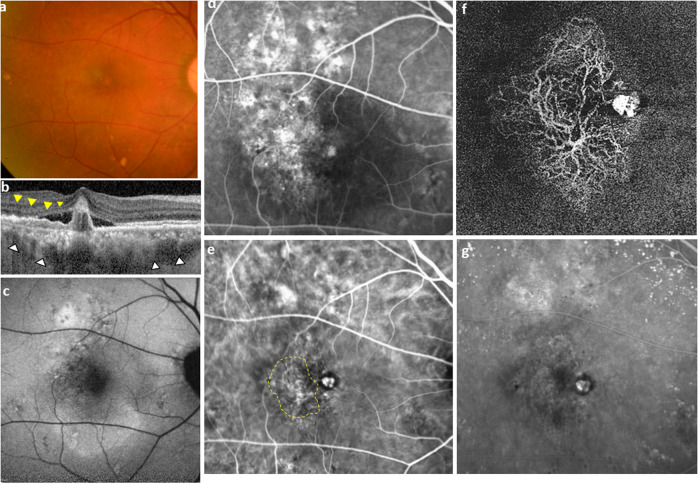
Multimodal imaging features of an eye with PCV. **a** Colour fundus photograph: A small orange nodule is visible close to the fovea. Several pachydrusen can be seen scattered in the outer macula. **b** Optical coherence tomography: a narrow-peaked pigment epithelial detachment (PED) can be seen in the centre of the OCT cut. Two hyporeflective ring-like structures can be seen inside the PED. The choroid appears thickened (white arrowheads), with most of the choroid occupied by large diameter Haller’s layer veins. A double-layer sign (yellow arrowheads) can be seen temporal to the narrow-peaked PED. **c** Blue-light fundus autofluorescence (FAF) image: widespread FAF disturbance can be seen, suggestive of retinal pigment epithelial disturbance. **d** Fluorescein angiography: occult leakage pattern is seen. This pattern is not helpful in differentiating PCV from type 1 macular neovascularization. **e** Early phase Indocyanine green angiography (ICGA): the polypoidal lesions (PLs) appear as nodular hyperfluorescent lesions corresponding to the orange nodule in (**a**) and the narrow-peaked PED in (**b**). The branching vascular network (BVN) is detected (yellow dotted outline) and dilated Haller’s layer veins can be seen. **f** OCT angiography: flow signal is detected in both the PL and the BVN. The appearance of the PLs appear as tangled vasculature on OCTA. **g** Mid-phase ICGA: the PLs display the typical appearance as nodular hyperfluorescent lesions. The BVN is not visualized in this timeframe. An area of hyperpermeable choroid corresponds to the area of dilated Haller’s veins in (**e**).

Due to the high prevalence of PCV in Asia, many academic centres in Asia perform ICGA as part of the routine investigations for all newly presenting exudative AMD cases. However, ICGA may not be routinely available, especially in smaller, non-academic settings. In these cases, it may be reasonable to only perform ICGA in cases showing suspicious signs of PCV. Similarly, in non-Asian populations, in which the prevalence of PCV is expected to affect one in five cases with exudative AMD, subjecting every patient to ICGA is probably not justifiable. There has been significant effort from many teams of researchers to identify non-ICGA based features which can help to differentiate PCV from typical nAMD. While FA is known to have low distinguishing power, combinations of OCT features have been reported by several groups. OCT features which have been suggested to be helpful include double-layer sign and thumb-like polyps, multiple pigment epithelial detachments (PED), sharp peaked PED, PED notch and a sub-RPE hyperreflective ring surrounding a hyporeflective halo within a PED [25, 60, 61]. Figure 1 shows an example of multimodal imaging features in PCV.

In 2018, the Asia-Pacific Ocular Imaging Society (APOIS) PCV workgroup was formed with the aim to promote the application of ocular imaging in the understanding and management of PCV worldwide. The first goal of the group was to develop and validate a set of non-ICGA criteria for differentiating PCV from typical nAMD. Based on published literature, the group selected 9 features (7 OCT-based, 2 colour fundus photograph-based), and evaluated and validated the accuracy of combination of these features against gold standard ICG in treatment-naïve eyes. Combination of 3 major criteria (sharp-peaked PED, sub-RPE ring-like lesion and complex multilobular PED on enface OCT) achieved an area under curve (AUC) of 0.9, positive predictive value of 0.93 and negative predictive value of 0.68 in differentiating PCV from typical nAMD [62]. Applying this set of OCT-based criteria can highlight cases with high likelihood of harbouring PCV in settings where ICGA is not routinely performed.

In a follow-up study, the group further evaluated non-ICGA features for differentiating PCV from typical nAMD in eyes with persistent fluid after initial anti-VEGF therapy. Presence of sub-RPE ring-like lesion, peaked-PED and orange nodule was able to differentiate eyes with active PCV from typical nAMD with an AUC of 0.85 [63]. When these criteria are present in eyes with persistent fluid, a few options to consider include switching anti-VEGF agent, shortening retreatment interval or additional monthly loading, or adding occlusive therapy. If ICG is not available, the PDT spot size can be planned based on RPE elevation on OCT. To assess whether polypoidal lesions have closed, we report densely hyperreflective PED content, absence of SRF and absence of sub-RPE ring with a combined AUC of 0.9 to differentiate closed versus perfused polypoidal lesions [64]. This set of criteria also has advantage over ICGA in differentiating PCV from other conditions that appear as hot spots (pseudo-polyps) on ICGA, such as Type 3 neovascularization and retinal macroaneurysm.

### Treatment- how would treatment outcome differ if a PCV case is treated as typical nAMD?

As discussed in previous sections, recent randomized trials and clinical experience have increasingly demonstrated that anti-VEGF monotherapy can achieve substantial visual acuity gain and drying of the retina in the majority of PCV cases. Nonetheless, a subset of PCV eyes may show suboptimal response to anti-VEGF monotherapy. From the pivotal clinical trials such as EVEREST II and PLANET, the proportion losing 15 letters or more was less than 10% [55, 56]. This proportion is similar to that reported with anti-VEGF for typical nAMD, such as in the MARINA, ANCHOR and VIEW studies [2, 3, 65]. PLANET offers a further opportunity to estimate the proportion of suboptimal responders to IVA monotherapy. Seventeen percent of the study cohort met pre-specified rescue criteria (BCVA ≤ 73 letters, and BCVA gain from baseline <5 letters or ≥5 but <10 letters and the investigator considered PDT could be beneficial, with new or persistent fluid on OCT and active polyp on ICGA) [56]. As such, the previous emphasis on the need to confirm the diagnosis of PCV at baseline with ICGA and initiate combination therapy has been reduced. Instead, the APOIS diagnostic criteria based on OCT can be used for screening purpose [62]. If the three major criteria are met, it is highly suggestive the case is PCV (positive predictive value 93%). While either anti-VEGF monotherapy or combination can be used as initial therapy, anti-VEGF monotherapy is preferred in settings where PDT is not readily accessible [9]. It is crucial to reassess after the loading phase based on the initial response in these cases. It is expected that majority of these PCV cases will show good response to anti-VEGF as evidenced by improvement in visual acuity and drying up of intra- and sub-retinal fluid and resolution of any haemorrhage. In these cases, further treatment with anti-VEGF can be continued. However, if after loading phase, responses are suboptimal (lack of visual acuity improvement, lack of resolution of fluid, further haemorrhage), performing ICGA is recommended to clarify the diagnosis. Options for further management include switching anti-VEGF agent and/or combination with PDT.

On the other hand, if the three major criteria are not met, the case is unlikely to be PCV (negative predictive value 68%) and anti-VEGF therapy can be initiated confidently. However, as with any condition, it is important to assess treatment response and re-think the diagnosis in cases not responding as expected.

#### Treatment regimen and disease activity assessment

**Fig. 2:**
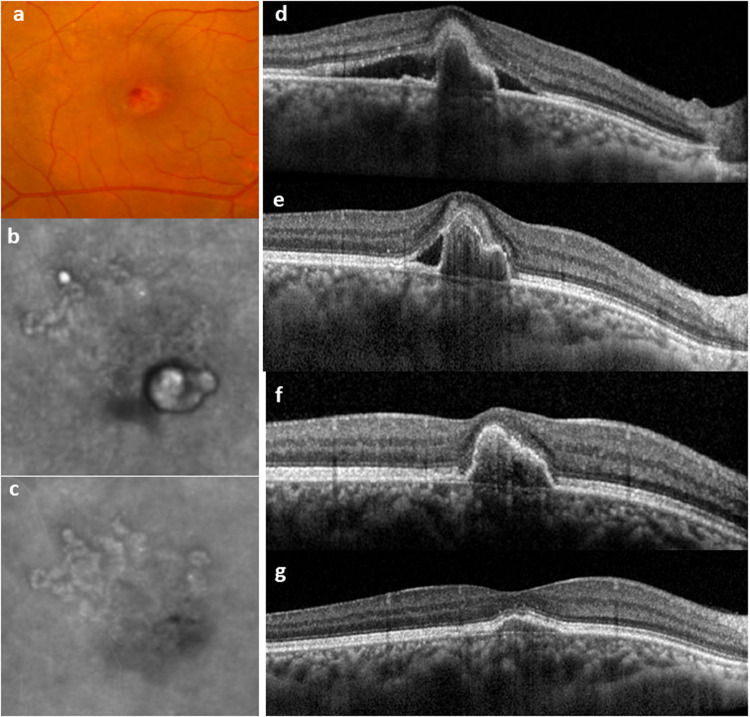
Example of an eye with PCV responding to anti-VEGF monotherapy. **a**, **b** Baseline colour fundus photo and ICGA showing a large polypoidal lesion. **c** Repeat ICGA at 1-year showing regression of the polypoidal lesions, while the BVN persists. **d**–**g** Sequential OCT from baseline, to month 3, 6 and 12 illustrating progressive resolution of subretinal fluid and flattening of PED. Thickened choroid can be seen throughout.

With the increasing acceptance of PCV as a variant of nAMD, the need for continued treatment as opposed to pro-re-nata treatment is also increasingly recognized. This shift further highlights the similarities between PCV and typical nAMD and the appreciation of the neovascular nature of the PCV network. The Asia Pacific Vitreo-retinal Society recently published consensus recommendations for treat-and-extend (T&E) regime in PCV which encompass many of the fundamentals of T&E [66]. Concurrently, imaging modality used for assessing disease activity has also shifted towards OCT-based (IRF, SRF) as opposed to ICGA based assessment of polyp closure. OCT fluid assessment, together with clinical assessment of visual acuity and presence of haemorrhage are the most commonly adopted markers for disease activity when applying a T&E strategies. In addition to IRF and/or SRF, the size and reflectivity of PED may also indicate whether polyps are perfused or closed following treatment. As the PCV complex is located below the RPE, an increase in PED size even in the absence of IRF or SRF should be considered an indicator of polyp reactivation in PCV cases. Figure 2 illustrates the response of an eye with PCV treated with anti-VEGF monotherapy.

#### Role for PDT

Combination therapy (PDT with anti-VEGF) continues to have a role in the management of PCV, particularly in the subgroup showing sub-optimal response to monotherapy. Combination therapy has been associated with earlier control of disease activity and a lower number of retreatments compared to monotherapy, therefore combination should be considered in patients who have difficulty in adhering to frequent injections [55, 67]. Certain phenotypes of PCV associated with aggressive disease, such as those with large area of polyps and those with hyperpermeable choroid, may also benefit from combination therapy [68–70]. The use of PDT however, is not without risk. These include post-treatment haemorrhage, choriocapillaris ischemia and damage to RPE. These risks increase with repeated PDT treatments.

## Recent progress in the understanding of pathogenic mechanisms

### International consensus nomenclature based on multimodal imaging findings

Correlation of multimodal imaging findings have led to improved understanding of the nature of the PCV lesion components. It is now widely accepted that the ‘polyp’ part of the lesion is vascular in nature as opposed to being a fleshy, solid mass [54, 71]. Specifically, the lesion can be seen to initially fill with dye, then pool and occasionally pulsate on dynamic angiography. Hence, some researchers have proposed to update the terminology for this lesion component to ‘aneurysmal lesion’. However, controversies remain regarding the structure of the vascular tissue within the ‘polyp’, with some researchers observing tangled vasculature rather than aneurysmal dilatation based on OCTA [72]. The APOIS PCV workgroup consensus nomenclature publication discussed these considerations and proposed the term ‘polypoidal lesion’ (PL) to replace ‘polyp’ until better understanding of the internal architecture of this lesion. Advances in the understanding of the BVN have also resulted in fundamental changes in the understanding of this lesion component [62]. Early studies based on ICGA lacked the ability to resolve depth that later studies with OCT offer. As such early studies proposed that the BVN may be intrachoroidal channels. However, OCT studies, supported by histopathologic studies, have indicated this network is located between Bruch’s membrane and the RPE, and therefore represents a form of neovascularization. Further observations of leakage and exudation arising from this network, and its persistence even after closure of polypoidal lesion are consistent with a neovascular network. The APOIS PCV workgroup recommended updating the term ‘branching vascular network’ to ‘branching neovascular network’ to emphasize the neovascular nature of this lesion component [62]. Understanding of these fundamentals have helped to de-mystify the nature of PCV, and help clinicians formulate management strategies accordingly.

### Pachychoroid as a novel disease causing mechanism

Drusen is one of the hallmarks of AMD and signify chronic RPE damage. However, PCV eyes have been consistently shown to have lower prevalence of drusen compared to eyes with typical nAMD. [12–15, 73] This discrepancy has been one of the key reasons behind the hypothesis that PCV is a distinct entity to AMD. Recently researchers have proposed that in addition to drusen-driven mechanisms, choroidal neovascularization may also arise from pachychoroid-driven mechanism, with the latter being more common in Asian populations. In pachychoroid eyes, a newly-described sub-phenotype of drusen has been described. Pachydrusen, differ from conventional soft drusen in their shape, distribution, and pattern of aggregation, and are associated with increased Haller’s layer thickness and attenuated choriocapillaris layer [74–76]. Pachydrusen may represent early stage RPE disturbance in relation to the pachychoroid phenotype, similar to pachychoroid pigment epitheliopathy, a mild form of disease within the pachychoroid spectrum. The significance of pachydrusen however, is not fully understood. It has been suggested that choroidal thickness may act as a modulator in the expression of AMD. In cross sectional studies, eyes with thin choroid typically exhibit the pseudodrusen and type 3 MNV subtypes during the non-exudative and the exudative stages of AMD, respectively. In contrast, eyes with thick choroid are associated with pachydrusen and PCV in the non-exudative and exudative stages of AMD, respectively. In a longitudinal study, we further observed that when eyes with pachydrusen developed exudative MNV the predominant phenotype was PCV [77]. However, unlike soft drusen, eyes with pachydrusen that progress to exudative MNV do not exhibit rapid expansion. These findings suggest that pachydrusen may be an epiphenomenon, a manifestation of the effects resulting from choroidal vasculature alteration.

**Fig. 3:**
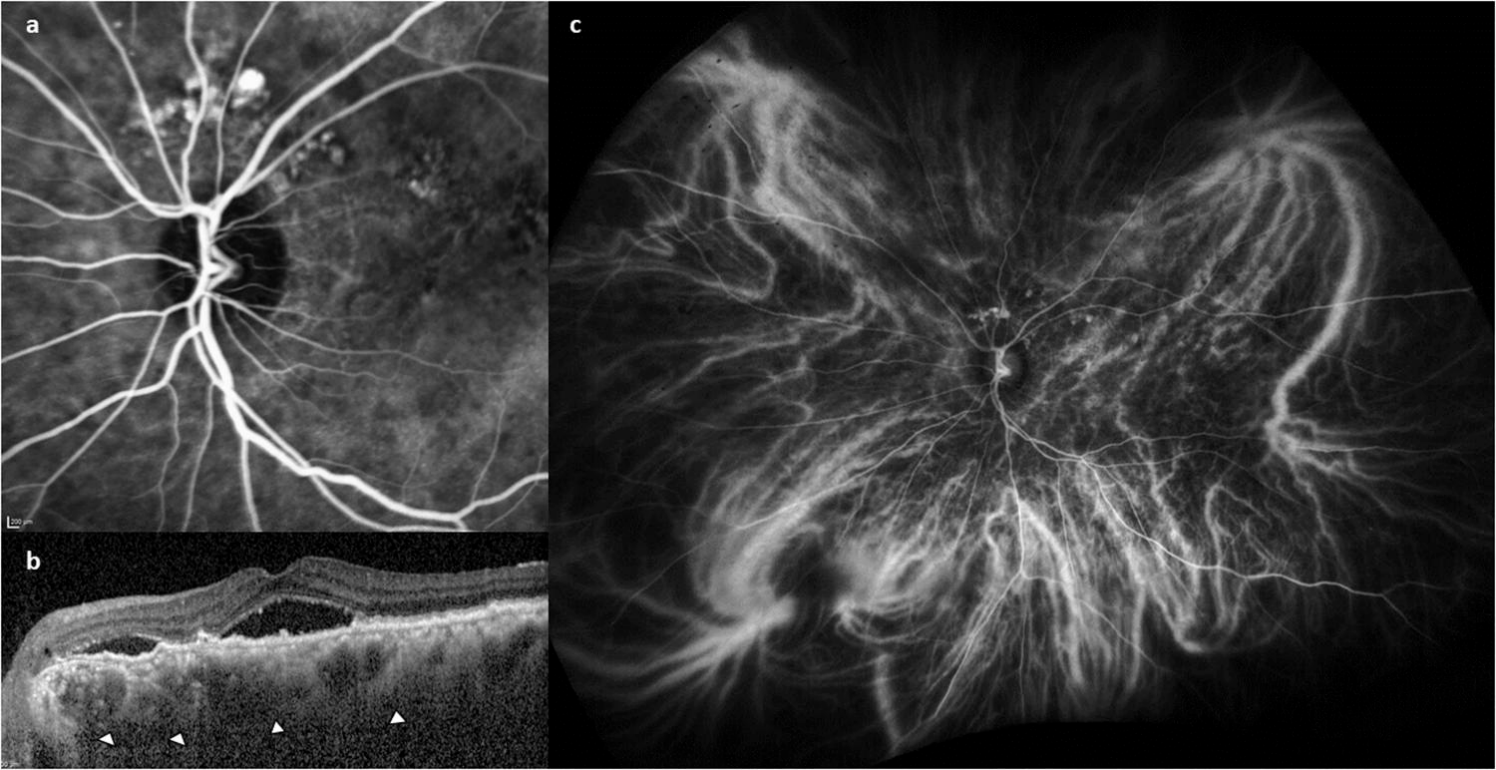
Choroidal alterations associated with PCV. **a** Multiple polypoidal lesions are detected superior to the optic disc on ICGA. **b** The OCT through the fovea shows presence of subretinal fluid and double layer sign. **c** On widefield ICGA, dilated Haller’s layer veins can be seen. Some of these dilated veins traverse the macular area and violate the horizontal watershed zone. Haller’s veins connecting the superotemporal and inferotemporal quadrants can be seen, suggesting these vessels may be anastomosis that develop during choroidal remodelling.

PCV is considered to reside within the pachychoroid spectrum [78]. Pachychoroid is a relatively new concept characterized by abnormally dilated Haller’s layer veins, choroidal hyperpermeability and often increased choroidal thickness. While the understanding of this concept continues to evolve with new observations, a significant body of work have been published in the past 1–2 years. Based on ultra-widefield ICG, we report that the dilated Haller’s layer veins are not simply dilated, but represent anastomosis between vortex vein systems [79, 80]. These can be seen to violate physiological watershed zones which follow the quadrantic arrangement of vortex vein outflow in normal. Figure 3 shows the widefield ICGA of an eye with peripapillary PCV. Furthermore, dynamic ICGA studies revealed abnormal pulsation suggestive of retrograde flow in segments of dilated Hallers vein, implying downstream obstruction [81]. The site where the vortex veins exit the eye via the sclera has been proposed to be a potential site for obstruction. Supporting this hypothesis, eyes with thicker sclera and shorter axial length have been associated with pachychoroid [82–84]. Importantly, the concept of a ‘pachychoroid’ phenotype has been proposed to be a novel mechanism that contributes to the pathogenesis of PCV through choriocapillaris ischemia. Filling delays in the choriocapillaris in the early phase of ICGA are frequently observed in PCV, which suggests choriocapillaris impairment [78]. OCTA studies have also reported increase in flow voids, suggestive of choriocapillaris impairment in eyes with pachychoroid phenotype. Although the mechanisms leading to choriocapillaris impairment is not fully understood, several potential mechanisms have been propose. These include: (1) compression of the choriocapillaris by pachyvessels (2) venous insufficiency and stasis leading to an ischaemic environment, and (3) primary choriocapillaris loss.

With increasing RPE and choriocapillaris damage resulting from the outer choroidal venous disturbance, neovascular complications develop at the advanced end of the pachychoroid disease spectrum. Pachychoroid neovasculopathy (PNV) has been proposed to be a potential precursor of PCV [85]. While longitudinal studies are limited, Siedlecki et al. reported in a longitudinal follow up study (mean 3.4 years) that PCV developed in 5 of 37 (13.5%) eyes with PNV [86].

### Comparison of PCV phenotypes between populations

There have been limited comparative studies of PCV features in Asians compared to non-Asians. These studies have reported PCV eyes among non-Asians have thinner choroid and higher frequency of drusen compared to PCV eyes among Asians [87, 88]. These features may reflect differences in the pathogenic mechanisms involved in different populations. In addition, a higher prevalence of subretinal haemorrhage and larger area of polypoidal lesions have also been reported in Asians compared to non-Asians. These differences may have implications on therapeutic response and should be further evaluated.

## Future research direction

Advances in imaging technology and the ability to visualize detailed structural alterations have been instrumental in driving the evolution in our understanding of PCV since its first description over 30 years ago. With the recent explosion in interest in the study of choroidal vascular alteration in pachychoroid and in PCV, coupled with advances in imaging technology, it would not be surprising if the next big leap in PCV may come in the form of choroid-related phenotyping and choroid-guided treatment strategies. Many groups are already working on quantifying choroidal vascular volume based on choroidal vascularity index or OCTA, and incorporating of artificial intelligence in image analysis [89, 90]. The ability to quantify choroidal congestion will enable researchers to evaluate the prognostic value of this new biomarker. Meanwhile, several key areas of research should be high on the agenda of the research community:

1. Clarification of definition and nomenclature around ‘pachychoroid’ concept, including longitudinal studies to understand evolution within the pachychoroid disease spectrum
2. Leverage on increasingly powerful imaging tools for deep phenotyping, focus on choroid and polypoidal/ aneurysmal lesions
3. International collaborations to understand the influence of ethnicity, genetics and epigenetics
4. Develop choroidal-guided treatment strategies

In conclusion, this article summarizes the progress in our understanding of PCV over the past 30 years. This journey has been driven by careful clinical observations leading to the recognition of phenotypic variations. Questioning the reason behind these differences have led to discovery of novel mechanisms. Ultimately, improved precision in phenotypes has led to more disease relevant management strategies and better outcome for patients.

## Summary

### What is known about this topic

- Differences in clinical and imaging features in PCV compared to typical neovascular AMD.

### What this study adds

- Summary of non-ICGA based diagnostic features.
- Summary of data which have driven the increasing adoption of anti-VEGF monotherapy in PCV.
- How choroidal alterations may contribute towards the development of PCV.
